# Targeting non-canonical activation of GLI1 by the SOX2-BRD4 transcriptional complex improves the efficacy of HEDGEHOG pathway inhibition in melanoma

**DOI:** 10.1038/s41388-021-01783-9

**Published:** 2021-05-06

**Authors:** Silvia Pietrobono, Eugenio Gaudio, Sinforosa Gagliardi, Mariapaola Zitani, Laura Carrassa, Francesca Migliorini, Elena Petricci, Fabrizio Manetti, Nikolai Makukhin, Adam G. Bond, Brooke D. Paradise, Alessio Ciulli, Martin E. Fernandez-Zapico, Francesco Bertoni, Barbara Stecca

**Affiliations:** 1 Core Research Laboratory – Institute for Cancer Research and Prevention (ISPRO), Florence, Italy; 2 Institute of Oncology Research, Faculty of Biomedical Sciences, USI, Bellinzona, Switzerland; 3 Department of Biotechnology, Chemistry and Pharmacy, University of Siena, Siena, Italy; 4 School of Life Sciences, Division of Biological Chemistry and Drug Discovery, University of Dundee, James Black Centre, Dundee, UK; 5 Schulze Center for Novel Therapeutics, Division of Oncology Research, Department of Oncology, Mayo Clinic, Rochester, MN USA; 6 Oncology Institute of Southern Switzerland, Bellinzona, Switzerland

**Keywords:** Melanoma, Cell signalling

## Abstract

Despite the development of new targeted and immune therapies, the prognosis of metastatic melanoma remains bleak. Therefore, it is critical to better understand the mechanisms controlling advanced melanoma to develop more effective treatment regimens. Hedgehog/GLI (HH/GLI) signaling inhibitors targeting the central pathway transducer Smoothened (SMO) have shown to be clinical efficacious in skin cancer; however, several mechanisms of non-canonical HH/GLI pathway activation limit their efficacy. Here, we identify a novel SOX2-BRD4 transcriptional complex driving the expression of *GLI1*, the final effector of the HH/GLI pathway, providing a novel mechanism of non-canonical SMO-independent activation of HH/GLI signaling in melanoma. Consistently, we find a positive correlation between the expression of GLI1 and SOX2 in human melanoma samples and cell lines. Further, we show that combined targeting of canonical HH/GLI pathway with the SMO inhibitor MRT-92 and of the SOX2-BRD4 complex using a potent Proteolysis Targeted Chimeras (PROTACs)-derived BRD4 degrader (MZ1), yields a synergistic anti-proliferative effect in melanoma cells independently of their *BRAF, NRAS*, and *NF1* mutational status, with complete abrogation of *GLI1* expression. Combination of MRT-92 and MZ1 strongly potentiates the antitumor effect of either drug as single agents in an orthotopic melanoma model. Together, our data provide evidence of a novel mechanism of non-canonical activation of GLI1 by the SOX2-BRD4 transcriptional complex, and describe the efficacy of a new combinatorial treatment for a subset of melanomas with an active SOX2-BRD4-GLI1 axis.

## Introduction

Melanoma is the most aggressive form of skin cancer and its incidence is increasing worldwide. Genetic alterations in *BRAF* and *NRAS*, as well as a handful of tumor suppressors such as *NF1, CDKN2A, ARID2* and *PTEN*, have been shown to contribute to melanoma pathogenesis [1, 2]. Aberrant activation of oncogenic BRAF has provided the basis for targeted therapy with specific inhibitors of mutant BRAF and MEK, although the long-term clinical benefits of these treatments are hampered by the development of drug resistance. Immune checkpoint inhibitors have shown more durable responses, although response rate still remains low [3]. Therefore, there is a need for novel treatments for relapsed or refractory melanoma patients based on new knowledge driving advanced stages of the disease.

Canonical Hedgehog/GLI (HH/GLI) signaling is triggered by binding of HH ligands to the twelve-pass transmembrane receptor Patched 1 (PTCH1). As such, PTCH1 no longer represses the seven-pass transmembrane G protein-coupled receptor Smoothened (SMO), allowing the intracellular activation of the zinc finger transcription factor GLI2, which translocates into the nucleus and transactivates *GLI1* promoter. Aberrant activation of HH/GLI signaling occurring in a variety of cancers leads to the activation of GLI transcription factors, which initiate and promote tumor growth by continuous transactivation of HH target genes [4]. Several studies have also reported non-canonical mechanisms of GLI activation in cancer, which may occur independent of upstream PTCH/SMO signaling [5]. Small molecule inhibitors targeting SMO have demonstrated therapeutic efficacy in advanced basal cell carcinoma (BCC) [6]. However, the successful clinical use of SMO antagonists is challenged by development of acquired resistance, severe adverse effects and relapse of patients upon drug withdrawal. Preclinical studies have shown the efficacy of SMO inhibition in reducing tumor burden in melanoma [7–9]. For example, the potent acylguanidine derivative targeting SMO (MRT-92) has shown good results in decreasing human melanoma xenograft growth in vivo [10]. Although MRT-92 appears a promising candidate for future clinical studies, interference with SMO alone may not be effective in blocking HH signaling in cancers having canonical and non-canonical HH/GLI signaling activation, such as melanoma. Only targeting non-canonical HH/GLI pathway is predicted to improve the response rates and durability of therapeutic effects exerted by SMO inhibitors. Thus, it is critical to investigate mechanisms of HH/GLI pathway activation downstream of SMO, especially those occurring at the transcriptional level.

In this study, we identify a novel BRD4-SOX2 transcriptional complex responsible for non-canonical activation of GLI1 in melanoma. We provide evidence that the chromatin reader BRD4 [11, 12] acts as cofactor of SOX2 to control *GLI1* promoter activity and expression. Combination of MZ1, a potent BRD4 degrader designed using the Proteolysis Targeted Chimeras (PROTACs) technology [13, 14], with the SMO inhibitor MRT-92 yields a synergistic reduction of melanoma cell growth in vitro and in vivo, providing a rationale for a novel therapeutic approach in melanoma.

## Results

### SOX2 modulates HH/GLI signaling by inducing non-canonical activation of GLI1

**Fig. 1:**
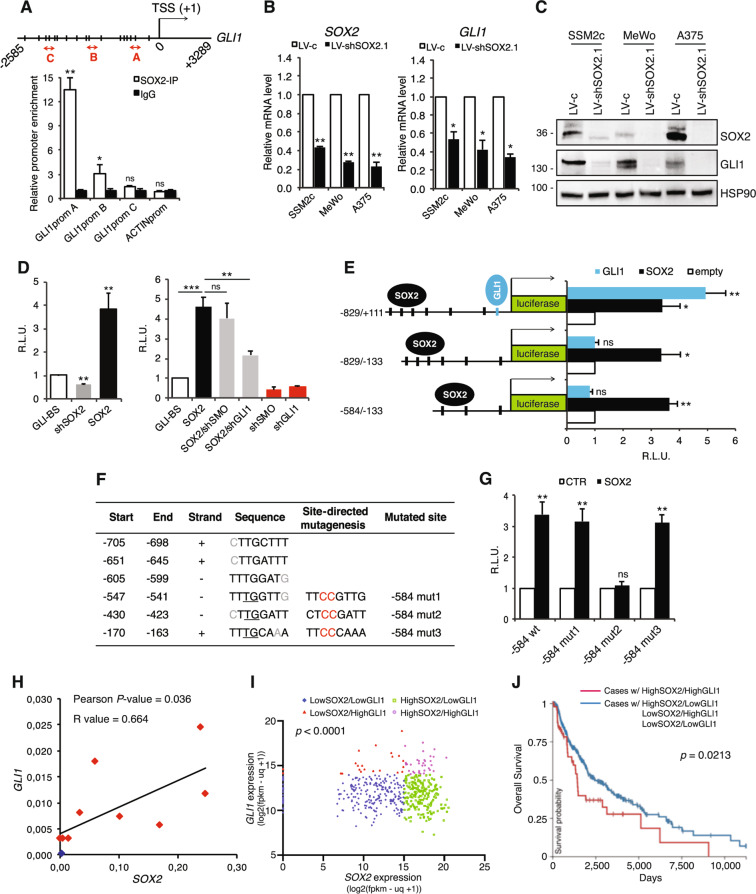
SOX2 binds to and transactivates *GLI1* promoter. **A** ChIP-qPCR of SOX2 occupancy at *GLI1* promoter (*n* = 3). Schematic representation of *GLI1* promoter with the position of ChIP probes (red double arrowhead) and consensus SOX2 binding sites (BS) (vertical slashes) relative to the transcription starting site (TSS). **B,C** qPCR (**B**) and Western blot (**C**) in melanoma cells transduced with LV-c or LV-shSOX2.1, showing that SOX2 silencing inhibits GLI1 expression in melanoma cells (*n* = 3). HSP90 was used as loading control in (**C**). **D** Dual-luciferase assay in SSM2c melanoma cells showing the effect of SOX2 modulation on the transactivation of a GLI-BS luciferase reporter (left). Silencing of GLI1, but not of SMO, is able to counteract SOX2-induced GLI-BS transactivation (right) (*n* = 4). **E** Dual-luciferase assay in SSM2c cells transfected with three different *GLI1* promoter fragments (−829/+111, −829/−133,−584/−133). It shows that SOX2 is able to transactivate all three promoter regions (*n* = 5). **F** Putative SOX2-BS in the −829 bp *GLI1* promoter with mutagenized sites (Mut1, Mut2 and Mut3). **G** Dual-luciferase assay in SSM2c cells showing that Mut2 prevented SOX2 from transactivating the −584 bp fragment of the *GLI1* promoter (*n* = 3). **H** Pearson correlation analysis of *GLI1* and *SOX2* mRNA in normal human epidermal melanocytes (NHEM, blue) and human melanoma cells (red) (*p* = 0.036). **I** Scatter plot of GLI1/SOX2 expression, where each data point represents an individual case. Graph was generated in Prism using data from the TCGA Melanoma (SKCM) dataset. Kruskal–Wallis test was used to compare four groups (*p* < 0.0001). **J** Overall Survival curve of cases from the SKCM Melanoma dataset in TCGA. Red line represents cases that have high expression of both *SOX2* and *GLI1*, and blue line represents cases that do not. The plot was generated using Xena software. The two curves were compared using Log-rank test (*p* = 0.0213). In (**A**, **B**, **D**, **E**, **G**) data are presented as mean ± SEM. *P* values were calculated by two-tailed unpaired Student’s *t* test (**A**, **B**, **D** left panel, **E**, **G**) or one-way ANOVA with Tukey’s test (**D**, right panel). *, *p* < 0.05; **, *p* < 0.01; ***, *p* < 0.0001; ns not significant.

Transcriptional activation of GLI1 independent of upstream SMO is one of the major driver of non-canonical activation of the HH/GLI pathway [5]. In silico analysis of *GLI1* promoter (obtained from the UCSC Genome Browser assembly ID:hg38) (Supplementary Fig. 1) using the TFBIND bioinformatics software (http://tfbind.hgc.jp) revealed significant enrichment of binding motifs (wwTGnwTw) [15] for SOX2 (Fig. 1A), a well characterized transcription factor involved in stemness, drug resistance, and tumor growth [16]. Chromatin Immunoprecipitation (ChIP) using anti-SOX2 antibody followed by quantitative real-time PCR (qPCR) with primers spanning different regions of *GLI1* promoter revealed SOX2 occupancy close to GLI1 transcription start site (TSS) (region A) with more than tenfold enrichment in GLI1 signal over ChIP with an isotype IgG (*p* < 0.01) (Fig. 1A). To investigate the regulation of GLI1 by SOX2, we first tested the effect of SOX2 silencing on the expression of GLI1. Depletion of SOX2 using two independent shRNAs significantly decreased the expression of GLI1 mRNA and protein in several melanoma cell types (Fig. 1B and C; Supplementary Fig. 2). We next tested the effect of SOX2 modulation on the transcriptional activity of the endogenous HH/GLI signaling. While SOX2 silencing led to a significant reduction of GLI-dependent luciferase reporter activity, its ectopic expression strongly increased it (Fig. 1D). Silencing of GLI1, but not that of SMO, was able to partially revert the effect of SOX2 overexpression on the reporter activity, confirming that SOX2 regulates HH/GLI pathway downstream of SMO (Fig. 1D).

To identify the site responsible for the modulation by SOX2, we cloned the following regions of the *GLI1* promoter upstream a luciferase gene: the *GLI1* proximal promoter (−829/+111 bp from TSS) containing six potential SOX2 binding sites (BS) and a canonical GLI-BS close to TSS; the −829/−133 bp region of the *GLI1* promoter lacking the GLI-BS; and the −584/−133 bp region containing only three putative SOX-BS (Fig. 1E). All three promoters showed similar basal activity in melanoma cells (Supplementary Fig. 3). Patient-derived SSM2c melanoma cells were transfected with the reporter along with SOX2 or GLI1. Luciferase assay showed that SOX2 transactivated all three regions (Fig. 1E), excluding the involvement of GLI1 on self-transactivation and narrowing the presence of a functional SOX2-BS in the three sites proximal to the *GLI1* TSS. To precisely map the SOX2-BS, we mutated each consensus sequences identified in the −584/−133 bp fragment in two crucial positions for the efficiency of SOX2 binding (Fig. 1F). Disruption of site2 (−584 mut2), but not that of site1 (−584 mut1) nor site3 (−584 mut3) prevented the transactivation of *GLI1* by SOX2 (Fig. 1G), indicating that SOX2 transactivates *GLI1* by direct binding to the consensus sequence CTTGGATT in *GLI1* proximal promoter.

In support of the biological relevance of the transcriptional regulation of GLI1 by SOX2, we found a statistically significant correlation between *SOX2* and *GLI1* expression in a panel of metastatic melanoma cells (Pearson score *R* = 0.664, *p* = 0.036) (Fig. 1H; Supplementary Fig. 4a). Furthermore, comparison of *GLI1* and *SOX2* expression levels in 477 TCGA melanoma patients showed a co-expression of these two transcripts (*p* < 0.0001) (Fig. 1I) and a significant decrease in overall survival in cases with high expression of both *SOX2* and *GLI1* (*p* = 0.0213) (Fig. 1J). In addition, the subgroup of melanoma cases with high expression of both *SOX2* and *GLI1* exhibited lower frequency of KRAS mutations (0.0182 vs. 0.292) and higher frequency of PTEN mutations (0.1272 vs. 0.0414) (Supplementary Fig. 4b), although the relevance of this finding needs further investigation.

### BRD4 is required for binding and transactivation of *GLI1* promoter by SOX2

**Fig. 2:**
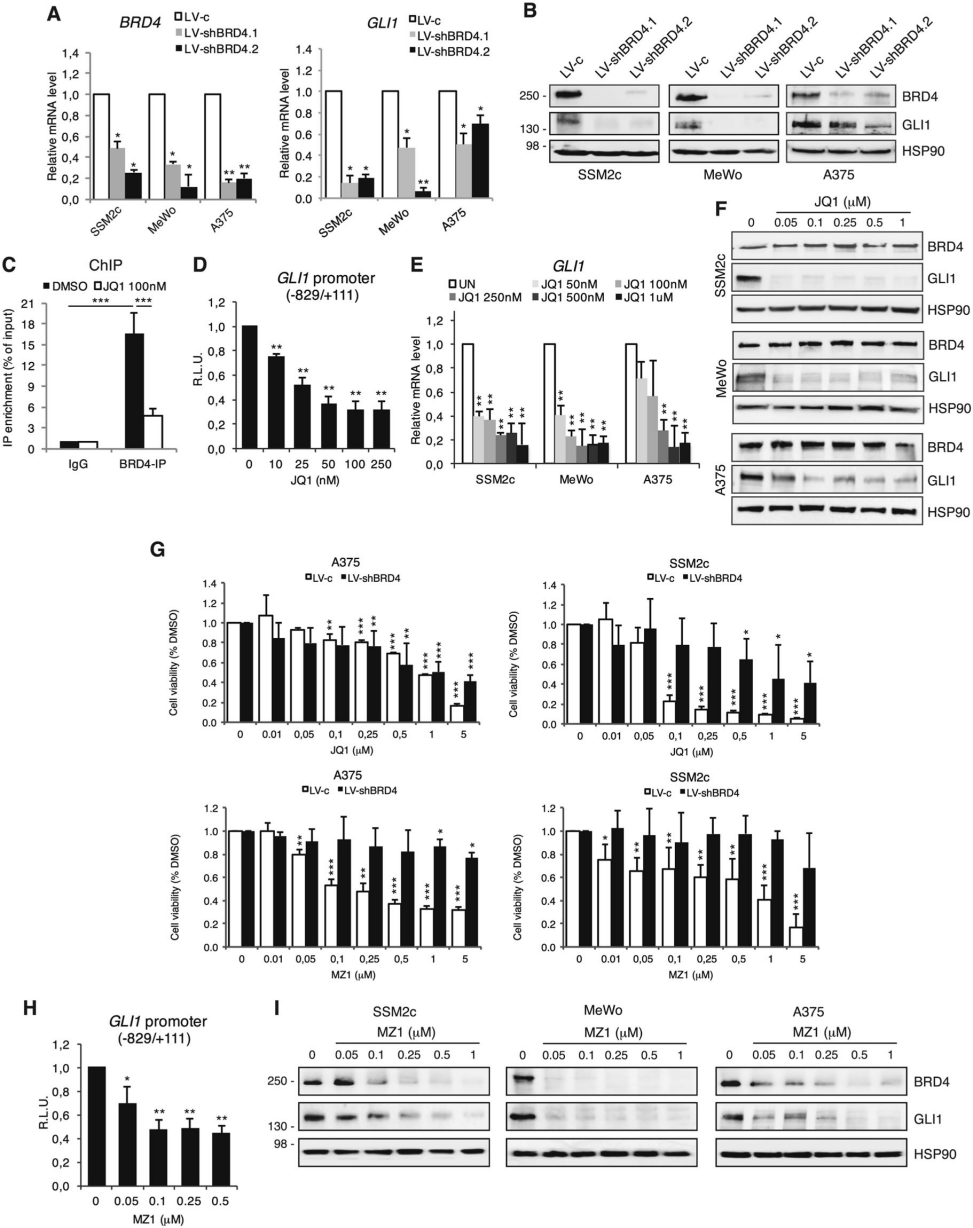
BRD4 regulates GLI1 transcription in melanoma. **A** qPCR of *BRD4* and *GLI1* after *BRD4* silencing with two independent shRNAs. **B** Western blot analysis of BRD4 and GLI1 in melanoma cells transduced as indicated. HSP90 was used as loading control. **C** ChIP-qPCR of BRD4 occupancy on *GLI1* promoter in SSM2c treated with vehicle (DMSO) or 100 nM JQ1 for 18 h. Data are presented as % of input and are expressed as fold over IgG control ± SEM (*n* = 3). **D** Quantification of dual-luciferase assay in SSM2c melanoma cells treated with DMSO or increasing concentrations of JQ1. Relative luciferase activities were firefly/Renilla ratios, with the level induced by the vehicle equated to 1 (*n* = 3). **E** qPCR of *GLI1* in three melanoma cell lines treated with DMSO or increasing concentrations of JQ1. **F** WB of BRD4 and GLI1 in melanoma cells treated with JQ1 as indicated. HSP90 was used as loading control. **G** Histogram of melanoma cell viability in cells transduced with LV-c or LV-shBRD4 and treated with DMSO or increasing concentrations of JQ1 or MZ1 (*n* = 3). **H** Quantification of dual-luciferase assay in SSM2c cells treated with DMSO or increasing concentrations of MZ1 (*n* = 3). **I** WB of BRD4 and GLI1 in melanoma cells treated with DMSO or increasing concentrations of MZ1. HSP90 was used as loading control. Data are presented as mean ± SEM. *P* values were calculated by two-tailed unpaired Student’s *t* test (**A**, **D**, **E**, **G**, **H**) or one-way ANOVA with Tukey’s test (**C**). *, *p* < 0.05; **, *p* < 0.01; ***, *p* < 0.0001.

The identified SOX2-consensus sequence within *GLI1* promoter is close to a DNA element showing enrichment of BRD4 [17, 18]. Since BRD4 is highly expressed in melanoma (Supplementary Fig. 4a) [19–22], we investigated its requirement in the binding and transactivation of *GLI1* promoter by SOX2. Silencing of BRD4 using two independent shRNAs (LV-shBRD4.1 and LV-shBRD4.2) strongly decreased the expression of GLI1 at both mRNA and protein level in melanoma cells (Fig. 2A, B). ChIP-qPCR of BRD4 confirmed occupancy of BRD4 at the *GLI1* promoter, with approximately 16-fold enrichment in GLI1 signal over ChIP with non-specific IgG (*p* < 0.05), which was abrogated upon pharmacological blockade of BRD4 activity with the pan-selective BET inhibitor JQ1 (Fig. 2C). JQ1 also led to a significant decrease of GLI1 transactivation, consistently to the robust decrease of GLI1 mRNA and protein (Fig. 2D–F). Our data are in line with previous reports showing that inhibition of BRD4 restrains HH/GLI-dependent growth of medulloblastoma, BCC, breast and pancreatic cancers [17, 18, 23, 24].

Although BET inhibitors show limited selectivity due to the inability to discriminate between the BD1 and BD2 bromodomains across the BET family members, which are overexpressed in melanoma cells, we confirmed our data with the recently developed MZ1, a PROTAC chimera that links JQ1 to a ligand for the E3-ubiquitin ligase VHL, rapidly inducing enduring and preferential removal of BRD4 over BRD2 and BRD3 [13, 14]. Indeed, BRD4 silencing nullified the effect of MZ1 on cell viability, but partially desensitized that of JQ1, confirming the higher specificity of MZ1 (Fig. 2G). To prove the selectivity of MZ1 toward BRD4 vs. BRD2 and BRD3, we monitored expression levels of BRD proteins in SSM2c, A375 and MeWo cells treated with increasing doses of MZ1. While MZ1 treatment induced a strong reduction (more than 50%) of BRD4 protein expression starting from 0.05 to 0.1 μM in all three cell lines, the expression of BRD2 and BRD3 proteins was affected only at higher doses (Supplementary Fig. 5a). Further, silencing of BRD2 or BRD3 did not change the effect of MZ1 on melanoma cell viability compared to control cells (Supplementary Fig. 5b–e). Taken together, our data indicate that MZ1 induces preferential degradation of BRD4 over BRD2 and BRD3. Notably, MZ1 led to a significant reduction of GLI1 transactivation with overall decrease of GLI1 levels, paralleling the effects observed after genetic silencing of BRD4 (Fig. 2H, I).

**Fig. 3:**
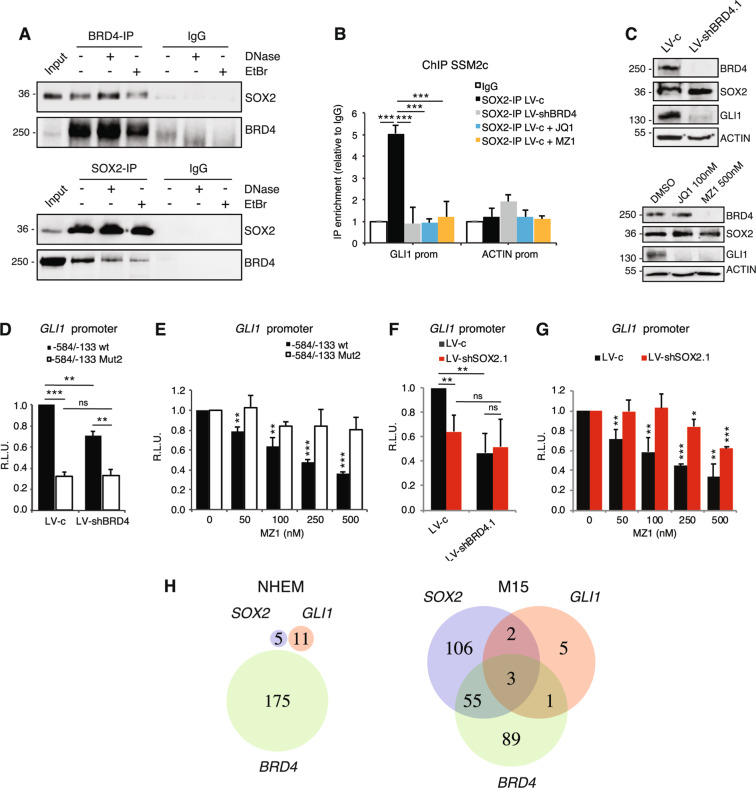
BRD4 acts as a SOX2 cofactor in GLI1 transcriptional activation. **A** Co-IP of SOX2 and BRD4 in SSM2c lysates untreated or exposed to 25 U/ml DNase or 200 μg/ml ethidium bromide (EtBr). Input was 5%. **B** ChIP-qPCR of SOX2 occupancy at *GLI1* promoter in SSM2c LV-c treated with vehicle (DMSO), JQ1 (100 nM) or MZ1 (125 nM), or LV-shBRD4. **C** Western blot of BRD4, SOX2 and GLI1 in SSM2c cells transduced with LV-c or LV-shBRD4 (upper panel) or treated with DMSO, JQ1 or MZ1 for 24 h (lower panel). **D**–**E** Dual-luciferase assay in SSM2c cells transduced with LV-c or LV-shBRD4 (**D**) or treated with increasing concentrations of MZ1 (**E**). It shows that Mut2 prevents BRD4 from transactivating the −584/−133 fragment of *GLI1* promoter in absence of a functional SOX2-BS (Mut2) (*n* = 3). **F**–**G** Dual-luciferase assay in SSM2c cells transduced with LV-c or LV-shBRD4 in presence or absence of SOX2 (**F**) or treated with increasing concentrations of MZ1 in presence or absence of SOX2 (**G**) as indicated (*n* = 3). **H** Venn diagram showing that the distribution of cells expressing *GLI1, SOX2*, and/or *BRD4* is different between normal human neonatal epidermal melanocytes (NHEM, left) and patient-derived melanoma xenografts (M15, right). Single cell RNA-seq data were filtered to the 80th percentile of high gene expression, and number of cells expressing one, two, or all three of these genes was quantified. The green circle represents number of cells expressing *BRD4*, the blue circle represents *SOX2*, and the red circle represents *GLI1*. The cutoff values for expression representing the 80th percentile in *SOX2, GLI1*, and *BRD4* were 0.06783216, 0.6914666, and 0.6897033, respectively. In (**B**, **D**, **E**, **F**, **G**), data are presented as mean ± SEM. *P* values were calculated by one-way ANOVA with Tukey’s test (**B**) or two-tailed unpaired Student’s *t* test (**D**–**G**). *, *p* < 0.05; **, *p* < 0.01; ***, *p* < 0.0001; ns not significant.

As BRD4 interacts with acetyl-lysine residues of histone 3 (H3) and histone 4 (H4) functioning as transcriptional coactivator [25], we next investigated whether BRD4 functions as SOX2-cofactor. We performed protein co-immunoprecipitation (Co-IP) of SOX2 and BRD4 in presence of DNase I, or ethidium bromide to unwind the DNA helix. Western blot showed that SOX2 and BRD4 proteins co-immunoprecipitated despite treatments (Fig. 3A), suggesting that interaction between these two proteins may be direct and independent from their interaction with neighboring regions of DNA. We then addressed the requirement of BRD4 for SOX2-induced transcriptional activation of GLI1. Silencing of BRD4, as well as its pharmacological depletion with MZ1 or catalytic inhibition through JQ1, prevented SOX2-binding to *GLI1* promoter (Fig. 2B, C), without altering SOX2 expression in melanoma cells (Supplementary Fig. 6). Both genetic and pharmacological inhibition of BRD4 led to a significant decrease of the luciferase activity in presence of the −584/−133 bp fragment of *GLI1* promoter containing a functional SOX-BS (wt) but failed to affect that of the promoter in which the SOX-BS was disrupted (Mut2) (Fig. 3D and E). To confirm this, genetic silencing of SOX2 almost completely abrogated the effect of BRD4 or MZ1 on the transactivation of GLI1 promoter (Fig. 3F, G). However, we cannot exclude that BRD4 could affect *GLI1* transcription by binding to additional regulatory regions of GLI1 (i.e., distal enhancers). Indeed, BRD4 silencing led to decreased GLI1 expression even in absence of SOX2, albeit to a lesser extent (Supplementary Fig. 7). Altogether these data indicate that BRD4 acts as a SOX2 cofactor to induce GLI1 transcriptional activation. To further support the relevance of this regulation during melanoma progression, single-cell analysis in cells derived from normal melanocytes and from a melanoma brain metastasis PDX model (M15) showed co-expression of *SOX2, BRD4* and *GLI1* in a subgroup of metastatic melanoma cells but not in melanocytes (Fig. 3H).

### The SMO antagonist MRT-92 synergizes with the BRD4 degrader MZ1 to inhibit melanoma cell growth

**Fig. 4:**
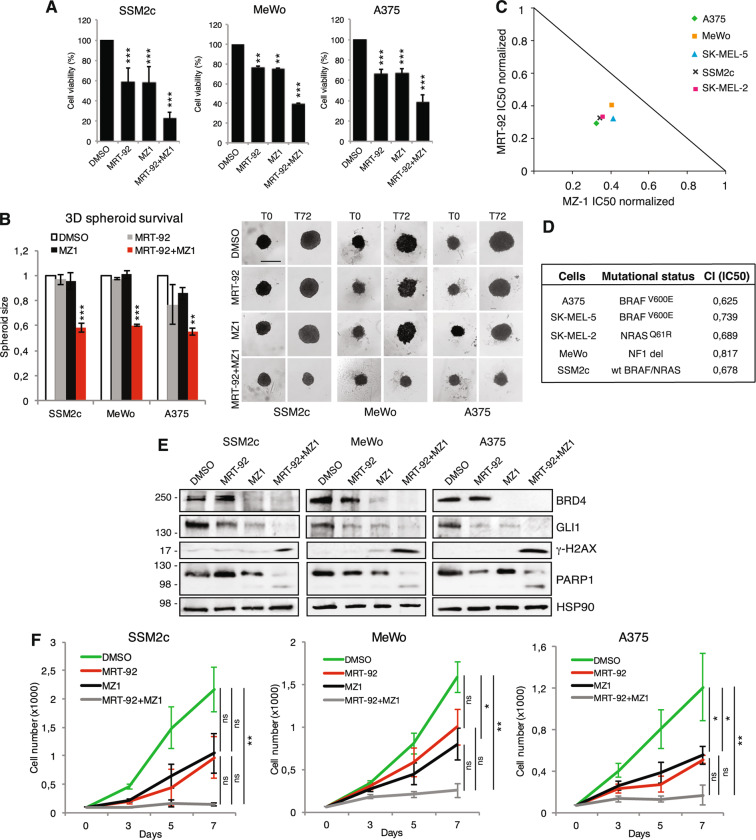
Co-targeting SMO and BRD4 reduces melanoma growth in vitro. **A** Histograms of melanoma cell viability after treatment with DMSO, MRT-92 (SSM2c and A375, 250 nM; MeWo, 300 nM), MZ1 (SSM2c and MeWo, 125 nM; A375 250 nM) or their combination for 72 h. **B** Histograms of 3D spheroid size at the optimized seeding densities (T0) or after 72 h of treatment as indicated in (**A**). Scale bars = 200 µm. **C** Normalized IC_50_ isobologram showing synergistic effects of MRT-92 and MZ1 combination. **D** Table showing the combination index (CI) values at the IC_50_ of MRT-92 and MZ1 with melanoma cell mutational status. **E** Representative WB of BRD4, GLI1, PARP-1 and γ-H2AX cells treated for 72 h as indicated (*n* = 3). HSP90 was used as loading control. In (**A**, **B**) data are presented as mean ± SEM. **F** Growth curves of melanoma cells treated for 7 days with MRT-92, MZ1 or combination at the following concentrations: SSM2c: MRT-92 at 250 nM and MZ1 at 125 nM; MeWo: MRT-92 at 300 nM and MZ1 at 125 nM; A375: MRT-92 at 250 nM and MZ1 at 250 nM. Data are expressed as fold percentage of vehicle (DMSO) ± SEM (*n* = 3). *P* values were calculated by one-way ANOVA with Tukey’s test (**A**, **B**, **F**). *, *p* < 0.05; **, *p* < 0.01; ***, *p* < 0.0001; ns not significant.

**Fig. 5:**
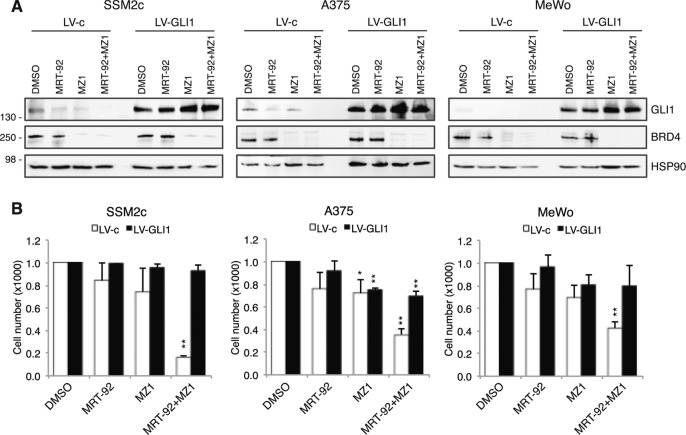
GLI1 overexpression rescues the effects of combined inhibition of SMO and BRD4. **A** Western blot of BRD4 and GLI1 in melanoma cells transduced with LV-c or LV-GLI1 after treatment with MRT-92, MZ1 or their combination for 72 h. HSP90 was used as loading control. **B** Cell viability of SSM2c, A375 and MeWo cells transduced with LV-c or LV-GLI1 after 72 h of treatment with MRT-92, MZ1 or combination at the following concentrations: SSM2c: MRT-92 at 250 nM and MZ1 at 125 nM; A375: MRT-92 at 250 nM and MZ1 at 250 nM; MeWo: MRT-92 at 300 nM and MZ1 at 125 nM. Data are expressed as fold change of vehicle (DMSO) ± SEM. (*n* = 3). *P* values were calculated by one-way ANOVA with Tukey’s test. *, *p* < 0.05; **, *p* < 0.01.

As specific inhibitors for SOX2 are currently not available, we investigated the therapeutic efficacy of combined SMO and BRD4 targeting. Co-administration of MRT-92 and MZ1 or JQ1 led to marked cytotoxic activity compared to single agents in melanoma cells grown either as a monolayer or as three-dimensional (3D) cell cultures (Fig. 4A, B; Supplementary Fig. 8). The isobologram summarizes the combination index (CI) at the IC_50_ when the SMO inhibitor MRT-92 and the BRD4-degrader MZ1 were combined, showing a moderate synergistic anti-proliferative effect (CI < 1) in melanoma cells independently of their *BRAF, NRAS*, and *NF1* mutational status (Fig. 4C, D). Western blot analysis of GLI1 confirmed that the two small molecules are variably effective in decreasing GLI1 protein level, and almost completely abrogated GLI1 when used in combination (Fig. 4E). MRT-92/MZ1 combination also appeared to induce signs of DNA damage, as shown by cleavage of poly ADP-ribose polymerase-1 (PARP-1) and increased phosphorylation of ɣH2AX (Fig. 4E). To investigate the durability of the effects of the drug combination, SSM2c, MeWo and A375 cells were treated with concentrations of MRT-92 and MZ1 that displayed a strong synergistic effect when combined, and cell growth was monitored up to 7 days without any additional drug administration. Growth curves showed that while single treatments barely affected melanoma cell growth, their combination almost abrogated it, indicating the long-lasting effect of the drug combination in inhibiting melanoma cell growth (Fig. 4F). Importantly, ectopic expression of GLI1 rescued the effect of MRT-92 and MZ1 combination in reducing melanoma cell viability (Fig. 5).

### Combined targeting of SMO and BRD4 suppresses self-renewal of melanoma stem-like cells

**Fig. 6:**
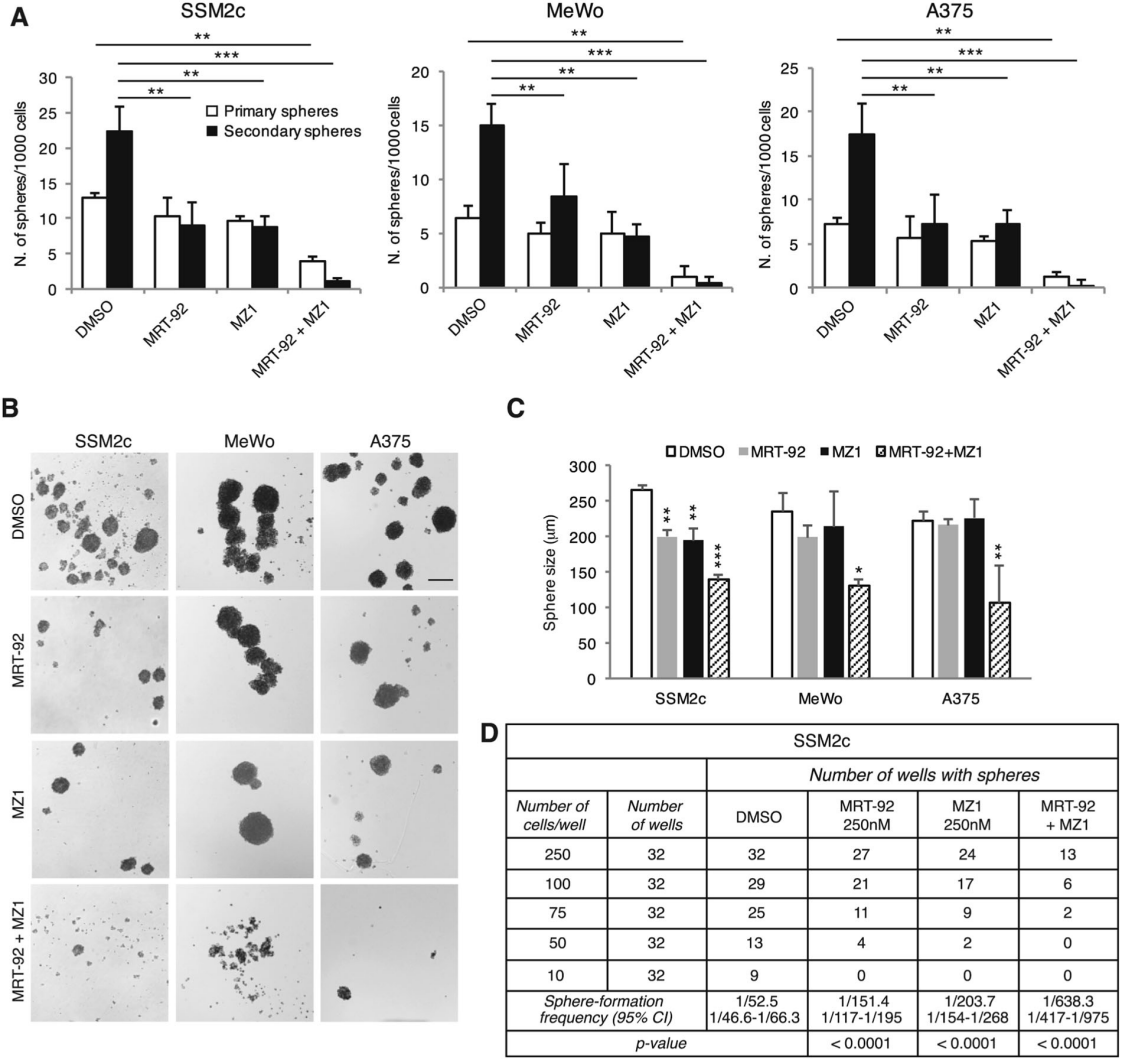
Combined targeting of SMO and BRD4 inhibits melanoma sphere self-renewal and survival. **A** Primary (white bars) and secondary (black bars) sphere formation assays from SSM2c, MeWo and A375 melanoma cells treated with MRT-92, MZ1 or combination (*n* = 3). **B** Representative phase-contrast images of secondary SSM2c, MeWo and A375 spheres as indicated in (**A**). Scale bar = 200 μm. **C** Size of secondary SSM2c, MeWo and A375 spheres treated as indicated. **D** Limiting dilution assays in SSM2c spheres. The observed average of sphere-forming frequency is shown, with the expected range reported below. In (**A**, **C**) data are presented as mean ± SEM. *P* values were calculated by one-way ANOVA with Tukey’s test. *, *p* < 0.05; **, *p* < 0.01; ***, *p* < 0.0001 vs. control.

We have previously shown that the HH/GLI signaling is critical for the maintenance of melanoma cancer stem-like cells (CSC) [26] whose occurrence has been shown to correlate with chemotherapeutic resistance, relapse and metastasis in several tumor types [27]. Other studies reported a role for BRD4 in promoting CSC self-renewal [28, 29]. Thus, we tested whether combined inhibition of SMO and blockade of BRD4 may synergize in reducing self-renewal ability of melanoma CSCs. Combined treatment with IC50 concentrations of MRT-92 and MZ1 significantly reduced primary sphere formation and suppressed their ability to self-renew and form secondary spheres (Fig. 6A, B). Secondary spheres were also reduced in size (Fig. 6C), suggesting an effect on proliferation or survival of committed or more-differentiated progenitors that make up the bulk of spheres.

To further address the effect of this drug combination on melanoma CSC maintenance, we performed limiting dilution assays (LDA) in SSM2c cells plated at varying densities. Results indicated that untreated control cells formed spheres at 250, 100, 75, 50 and 10 cells/well, with an estimated sphere-forming frequency of 1/52.5. Conversely, treatment with MRT-92 or MZ1 alone decreased sphere-forming capacity, dropping to 1/151.4 for MRT-92 (*p* < 0.0001) or 1/203.7 for MZ1 (*p* < 0.0001), whilst their combination almost abrogated sphere formation already at 75 cells/well dilution, with an estimated frequency of 1/638.3 (*p* < 0.0001) (Fig. 6D). Altogether, these data indicate that targeting both SMO and BRD4 drastically reduces the ability of melanoma-spheres to self-renew in vitro, supporting their efficacy against melanoma CSCs.

### Combined targeting of SMO and BRD4 shows a significant in vivo antitumor activity

**Fig. 7:**
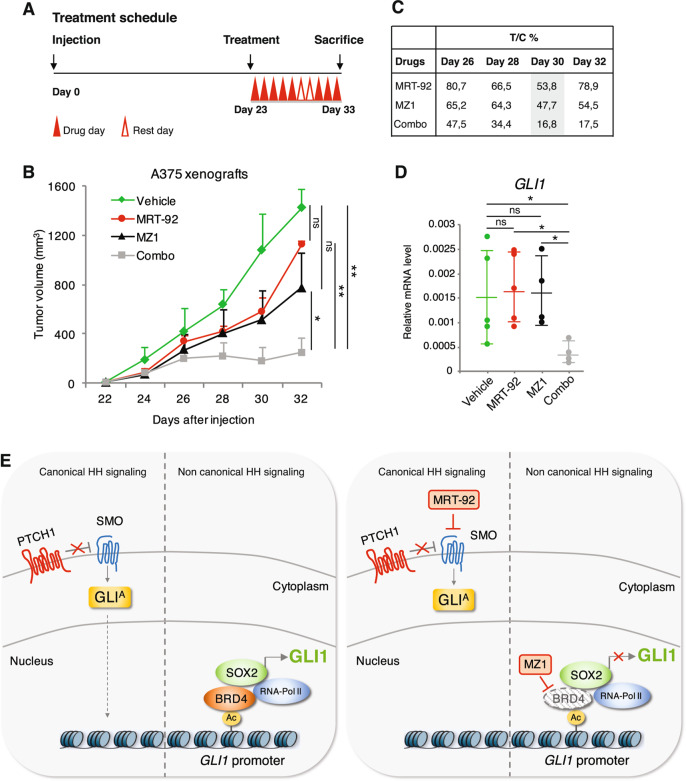
Efficacy of MRT-92 and MZ1 combination in vivo. **A** Schematic illustration of treatment schedule. **B** In vivo orthotopic tumor growth of A375 cells in athymic nude mice. At tumor appearance mice were randomized in four groups and treated i.p. with vehicle alone, MRT-92 (15 mg/Kg, BID), MZ1 (100 mg/Kg, QD), or combination (*n* = 7 for each group). **C** Table shows percentage of tumor volume reduction in treated groups compared to vehicle-treated group (% T/C ratio). **D** Dot plot quantification of *GLI1* expression by qPCR in A375 xenografts (*n* = 5 for each group). Data are presented as mean ± SEM. *P* values were calculated by ANOVA with Tukey’s test (**B**) or two-tailed unpaired Student’s *t* test (**D**). *, *p* < 0.05; **, *p* < 0.01; ns not significant. **E** Schematic representation of canonical and non-canonical HH signaling and their inhibition by MRT-92 and MZ1. Left, non-canonical activation of *GLI1* in melanoma: SOX2 and BRD4 form a complex, and BRD4, by interacting with acetylated histones in the proximal region of the *GLI1* promoter, induces RNA polymerase 2 activity and transcriptional activation of *GLI1*. Right, the SMO antagonist MRT-92 inhibits canonical HH signaling, whereas MZ1 induces BRD4 degradation with consequent inhibition of *GLI1* transcription.

To investigate the efficacy of the combined blockade of SMO and BRD4 in vivo, we assessed the effects of the drug combination in the growth of orthotopic A375 melanoma xenografts (Fig. 7A). No significant tumor growth inhibition was observed in animals treated with either MRT-92 (T/C% of 53.8) or MZ1 (T/C% of 47.7) as single agents, whereas a greater antitumor effect was achieved when combined (T/C% of 16.8) (Fig. 7B, C). Consistently, *GLI1* expression was completely abrogated only in tumors treated with the drug combination (Fig. 7D). Treatment with single agents or their combination was well tolerated in mice, without significant signs of toxicity. Throughout treatment, mice were well-conditioned with a body condition score [30] BC3 for all groups. Altogether, these results demonstrate that co-administration of MRT-92 and MZ1 improves the effect of single treatments against melanoma growth in vitro and in vivo.

## Discussion

Constitutive activation of GLI1 is associated with several types of cancer. Clinical trials based on the administration of SMO antagonists have demonstrated effectiveness in HH-driven tumors, such as BCC and medulloblastoma; however, the therapeutic efficacy of SMO inhibitors may not be effective in tumors having non-canonical activation of GLI1. Although many efforts in developing specific GLI inhibitors, good candidates for clinical trials are still lacking. Therefore, molecular inhibitors of GLI1 that directly affect its transcription by breaking the positive feedback loop may reveal very useful. In this study we show that SOX2 acts in a transcriptional complex with the epigenetic reader BRD4 to directly regulate GLI1 transcription, providing a novel mechanism of non-canonical SMO-independent activation of GLI1. The findings that MZ1 and MRT-92 combination completely abrogates GLI1 expression and that ectopic expression of GLI1 is able to rescue the inhibition of melanoma cell growth induced by the drug combination, suggest that GLI1 is the main molecular and functional target of the SOX2-BRD4 transcriptional complex. Therefore, as specific inhibitors of SOX2 are currently not available, targeting the SOX2-BRD4 transcriptional complex could be effective to curtail HH/GLI pathway downstream of SMO (Fig. 7E).

The existence of a direct regulation of SOX2 by GLI1 was previously described in melanoma, where both SOX2 and HH/GLI signaling are required for melanoma CSC self-renewal [26, 31]. This modulation has been associated with drug resistance in other types of cancer. For instance, GLI1-mediated regulation of SOX2 enhances CSC self-renewal and confers resistance to EGFR inhibitors in non-small cell lung cancer [32]. Accordingly, activation of the GLI-SOX2 axis is involved in gemcitabine resistance in pancreatic cancer [33]. Recently, a reciprocal regulation between SOX2 and GLI1 has been described to fuel aberrant glycosylation/sialylation during melanoma progression [34]. Altogether, these reports and the present study highlight the importance and the biological relevance of the mutual regulation between SOX2 and GLI transcription factors in cancer.

Previous studies have identified BET proteins as epigenetic regulators of HH transcriptional output, in particular of Gli1 and Gli2, and demonstrated that HH-driven tumors respond to JQ1 and I-BET151 [17, 18]. Chromatin immunoprecipitation (ChIP) coupled with DNA-sequencing have revealed highly asymmetric binding pattern of BRD4, with most chromatin bound BRD4 localized to super-enhancer elements important for cell-type specification and oncogenesis [35]. Whether super-enhancers are positioned over *GLI* promoters is still unknown, although our and other studies suggest that BRD4 occupies *GLI1* promoters [17, 18]. Previous reports have shown that BRD4 plays a critical role in melanoma [19, 20, 22, 36], representing a promising therapeutic target. However, the translational potential of pan-selective BET inhibitors used in these studies is limited. Indeed, the lack of discrimination between the BD1 and BD2 bromodomains across the BET family members could limit their selectivity and pose the threat of undesired side effects in clinical settings. Further, BET inhibitors such as JQ1 show a very short half-life, and the concentrations required to mediate single agent activity exceed physiologic safety levels in vivo [37]. The BRD4 degrader MZ1 potently and rapidly induces preferential removal of BRD4 over BRD2 and BRD3, and has shown high efficacy in ovarian and triple-negative breast cancer in vivo [38]. The activity of MZ1 is dependent on binding to VHL but is achieved at a sufficiently low concentration not to induce stabilization of the VHL substrate HIF-1α [13].

Here we show that co-targeting BRD4 and SMO elicits a significant antitumor activity in melanoma, including a drastic reduction of 2D and 3D melanoma cell growth and melanoma stem cell-like self-renewal. Notably, the efficacy of this combinatorial treatment in melanoma cells is not influenced by *BRAF, NRAS* or *NF1* mutational status, opening the possibility of using these compounds to treat melanoma expressing high levels of SOX2 and GLI1 irrespectively to their mutational status. This combinatorial treatment synergizes in inducing signs of DNA damage in melanoma cells, consistently with the role of BRD4 in promoting DNA repair [39], and with previous studies pointing to the induction of DNA damage following pharmacological inhibition of the HH/GLI signaling [10, 40]. Given that the efficacy of many antitumor agents relies on their ability to bypass DNA damage checkpoints with subsequent apoptosis [41], our findings could provide a valid alternative strategy to the current standard therapies.

The in vitro synergism translates into a marked antitumor activity in an orthotopic melanoma xenograft model. Indeed, the combination of MRT-92 and MZ1 almost completely abrogated in vivo tumor growth, despite the limited effect of single agents. Our data also indicate that SMO and BRD4 blockade is well tolerated in mice and does not cause any apparent side effect. Dual blockade of SMO and BRD4 might also contribute to prevent resistance to pan-selective BET inhibitors such as JQ1, because resistance to JQ1 can be mediated by GLI2-dependent upregulation of cMYC and targeting GLI2 restores JQ1 sensitivity in pancreatic cancer [42]. In addition, since BRD4 has been shown to enhance the escape of cancer cells from immunosurveillance through the regulation of the programmed death 1 (PD-1)/PD-L1 immune checkpoint [43–45], inhibition of BRD4 holds the potential to elicit an antitumor immune response. At this regard, the next-generation BET inhibitor PLX51107 was reported to delay melanoma growth in a syngeneic melanoma model by eliciting effects on anti-tumor CD8 + T cells [46].

In conclusion, in this study we provide evidence of a novel mechanism of non-canonical SMO-independent activation of GLI1 by the SOX2-BRD4 axis and describe the efficacy of a combinatorial treatment with a novel SMO inhibitor and the PROTAC-derived BRD4 degrader MZ1 in melanoma. The existence of a reciprocal regulation between SOX2 and GLI1 [31] (this study), which is involved in the transcriptional activation of genes involved in melanoma progression [34], highlights the therapeutic potential of targeting this axis to treat a subset of advanced melanomas expressing high levels of SOX2 and GLI1.

## Materials and methods

### Cell cultures

Normal human epidermal melanocytes (NHEM) and human melanoma cell lines A375, SK-Mel-2, SK-Mel-5, SK-Mel-28 and MeWo were obtained from ATCC, whereas A2058, 501-Mel and SK-Mel-197 were provided by Dr. Laura Poliseno (CNR, Pisa, Italy). Patient-derived metastatic melanoma cells SSM2c and M51 were already described [26, 47]. Cells were cultured in Dulbecco’s modified Eagle’s medium (DMEM), containing 10% fetal bovine serum (FBS), 1% penicillin-streptomycin solution (Lonza, Thermo Fisher Scientific) and 1% Glutamine (Lonza). All cells were authenticated by DNA fingerprinting analysis and regularly tested for potential Mycoplasma contamination.

### Compounds

The SMO inhibitor MRT-92 was already described [10]. The pan-BET inhibitor JQ1 (Catalog No. S7110, purity ≥99%) was purchased from Selleckchem (Munich, Germany). The PROTAC degrader MZ1 was synthesized in ~1 g scale by optimizing the previously described synthetic route [13].

### Quantification of the effect of the treatments

Crystal violet assay after 72 h treatment was used to measure cell proliferation using a plate reader (Victor X5, PerkinElmer). To obtain the response of cell lines to the combination of MRT-92 with either MZ1 or JQ1, cells were treated simultaneously with increasing concentrations of the two molecules. Results were examined by isobologram analysis with the Chou-Talalay Method by the Compusyn software [48] program to calculate the efficacy (CI) of the experimental points.

### Plasmids and viral production

Lentiviruses for gene silencing were produced in HEK-293T as previously described [10]. shRNA vectors used were: pLKO.1-puro (scramble, LV-c) (Addgene #8453), pLKO.1-puro-shSOX2.1 (LV-shSOX2.1) targeting the 3′ untranslated region of SOX2 (targeting sequence 5′-CTGCCGAGAATCCATGTATAT-3′), pLKO.1-puro-shSOX2.2 (LV-shSOX2.2) targeting the coding region of SOX2 (targeting sequence 5′-CAGCTCGCAGACCTACATGAA-3′) [31], pLKO.1-puro-shBRD4.1 (LV-shBRD4.1) targeting the coding region of BRD4 (targeting sequence 5′- CCTGGAGATGACATAGTCTTA-3′) and pLKO.1-puro-shBRD4.2 (LV-shBRD4.2) targeting the 3′ untranslated region of BRD4 (targeting sequence 5′-GCCAAATGTCTACACAGTATA-3′), pLKO.1-puro-shBRD2 (LV-shBRD2) targeting the 3′ untranslated region of BRD2 (targeting sequence 5′-CCCTTTGCTGTGACACTTCTT-3′), and pLKO.1-puro-shBRD3 (LV-shBRD3) targeting the 3′ untranslated region of BRD3 (targeting sequence 5′-CCAAGGAAATGTCTCGGATAT-3′). Lentiviruses for gene overexpression were produced in HEK-293T by co-transfection of CSGW vector, CSGW-SOX2 (cloned into the BglII-NotI restriction sites of CSGW vector using the following primers: SOX2-F 5′-ATGTACAACATGATGGAGACGG-3′ and SOX2-R 5′- TCACATGTGTGAGAGGGGC-3′) or CSGW-GLI1 (cloned into the BglII-NotI restriction sites of CSGW vector using the following primers: GLI1-F 5′-ATGTTCAACTCGATGACCCCAC-3′ and GLI1-R 5′-TTAGGCACTAGAGTTGAGGAA-3′) with pCMV-dR8.91 packaging plasmid and pMD2.G envelope plasmid (Addgene #12259).

### Chromatin immunoprecipitation

ChIP experiments were performed as previously described [34]. Primer sequences are listed in Supplementary Table 1.

### Quantitative RT-PCR

Quantitative real-time PCR was performed as already described [34].

### Mutagenesis and luciferase assay

Three fragments of *GLI1* promoter (−829/+111 bp, −829/−133 bp and −584/−133 bp) were PCR amplified with KOD hot start DNA polymerase (Merck Millipore) and cloned into the pGL3Basic vector (Promega) using NheI-HindIII sites, to generate −829/+111 bp, −829/−133 bp and −584/−133 bp GLI1 prom-luc reporters. Mutations of GLI1 prom −584/−133 reporter were introduced using QuickChange II (Agilent Technologies). All primers are listed in Supplementary Table 2. *GLI1* promoter reporters were used in combination with *Renilla* luciferase pRL-TK reporter vector (Promega) to normalize luciferase activities. Luminescence was measured using the Dual-Glo Luciferase Assay System (Promega) and the GloMax 20/20 Luminometer (Promega).

### Single-cell RNA sequencing

Melanoma patient-derived xenograft line M15 was derived from brain metastasis of Mayo Clinic patients under proper IRB and IACUC protocols. Normal human neonatal epidermal melanocytes (Lifeline Cell Technology) were used as a control. Single-cell RNA sequencing was performed at Mayo Clinic’s Genome Analysis Core Facility as already described [34]. Monocle3 [49] was used to analyze the single-cell RNA-seq dataset. Data are available from GEO under accession number GSE159597.

### Western blot and co-immunoprecipitation

Western blot and Co-IP were performed as already described [34]. List of primary antibodies is reported in Supplementary Table 3.

### Three-dimensional (3D) tumor-sphere assay

For 3D tumor-sphere generation, melanoma cells were plated in 1% FBS at optimal seeding densities (SSM2c 1500 cells/well, MeWo 3000 cells/well; A375 1500 cells/well) in ultra-low attachment (ULA) 96-well round bottom plates, centrifuged at 800 *rpm* for 3 min, allowed to form a three-dimensional structure within 24 h and photographed (T0). Spheroids were then treated with vehicle (DMSO), MRT-92 (250 nM for SSM2c and A375; 500 nM for MeWo), MZ1 (125 nM for MeWo; 250 nM for SSM2c and A375), JQ1 (100 nM for SSM2c and MeWo; 500 nM for A375) or combinations at the indicated concentrations for 72 h. Photos were executed with a LEICA DFC450C microscope with 4X objective lens, and both length and width of each spheroid measured using Image J, averaged and then normalized to that of T0.

### Melanoma-spheres and limiting dilution assay

SSM2c, MeWo and A375 melanoma-spheres were cultured in human embryonic stem cell medium supplemented with 4 ng/ml basic fibroblast growth factor. For primary sphere-formation and self-renewal, cells were plated in 12-well plates (Corning) at 1 cell/μl dilution, and allowed to form over 24 h before pharmacologic manipulation with vehicle (DMSO), MRT-92 (250 nM for SSM2c and A375; 500 nM for MeWo), MZ1 (125 nM for MeWo; 250 nM for SSM2c and A375), JQ1 (100 nM for SSM2c and MeWo, 500 nM for A375) or combinations for 96 h. Primary spheres were dissociated into single cells and re-plated at 1 cell/μl dilution in ULA 12-well plates. After 1 week, spheres were photographed and counted with a LEICA DFC450C microscope with 4X objective lens, and both length and width of each sphere were measured using Image J and averaged.

For limiting dilution assay, cells were plated at 250, 100, 75, 50 or 10 cell/well in sphere conditions in flat 96-well plates, and 32 wells per condition were assessed. Wells were scored positive (≥1 sphere/well) or negative (0 spheres/well) for sphere formation after 10 days in culture. Sphere forming frequency and statistics were calculated using ELDA software [50].

### Orthotopic melanoma xenografts

A375 melanoma cells were resuspended in Matrigel (Beckton Dickinson)/DMEM 1/1 and inoculated subcutaneously into the right lateral flank of adult (8 weeks) female athymic nude mice (Foxn1 nu/nu) (Charles River Laboratories) (10.000 cells/injection). Once tumors were palpable (≤100 mm^3^), mice were randomized in four groups and treated i.p. with MRT-92 (15 mg/Kg, BID), MZ1 (100 mg/Kg, QD), the combination of MRT-92 and MZ1 or the vehicle alone for 10 days. Both drugs were dissolved in vehicle (30% 2-hydroxypropyl-β-cyclodextrin) (Sigma-Aldrich). Subcutaneous tumor size was measured blindly three times a week with a caliper and tumor volume was calculated using the formula: V = W^2^ × L × 0.5, where W and L are tumor width and length, respectively. The Body Condition Scoring was used to assess mice health status [30]. No animals were excluded from the analysis. No statistical methods were used for sample size estimation. Mouse maintenance and animal experiments were performed according with the study protocol approved by the local Swiss Cantonal Veterinary Authority (No. TI-08-2019).

### The Cancer Genome Atlas analysis

The University of California Santa Cruz Xena platform was used to visualize and analyze transcriptomic and survival data from 477 cases in the TCGA melanoma (SKCM) cohort [51].

### Statistical analysis

Data represent mean ± SD or mean ± SEM values calculated on at least three independent experiments. No statistical methods were used for sample size selection. The estimate of variation within each group was similar. *P* values were calculated using Student’s *t* test (two groups) or one-way analysis of variance (ANOVA) (more than two groups). A two-tailed value of *p* < 0.05 was considered statistically significant. *, *p* < 0.05; **, *p* < 0.01, ***, *p* < 0.0001.

## Supplementary information

Supplementary Information
