# Supplementary material for: Targeting non-canonical activation of GLI1 by the SOX2-BRD4 transcriptional complex improves the efficacy of HEDGEHOG pathway inhibition in melanoma
Source: Oncogene. 2021 May 6;40(22):3799–814. doi: 10.1038/s41388-021-01783-9 (PMC8175236; doi:10.1038/s41388-021-01783-9)
Supplement: Supplementary file 1 — Supplementary Information [file 41388_2021_1783_MOESM1_ESM.docx]

**SUPPLEMENTARY INFORMATION**

**Targeting non-canonical activation of GLI1 by the SOX2-BRD4 transcriptional complex improves the efficacy of HEDGEHOG pathway inhibition in melanoma**

Silvia Pietrobono^1*^, Eugenio Gaudio^2^, Sinforosa Gagliardi^1^, Mariapaola Zitani^1^, Laura Carrassa^1^, Francesca Migliorini^3^, Elena Petricci^3^, Fabrizio Manetti^3^, Nikolai Makukhin^4^, Adam G. Bond^4^, Brooke D. Paradise^5^, Alessio Ciulli^4^, Martin E. Fernandez-Zapico^5^,

Francesco Bertoni^2,6^, Barbara Stecca^1,*^

**SUPPLEMENTARY MATERIALS AND METHODS**

**Treatments**

Compounds were used at the indicated concentrations and time in low serum conditions (1% FBS for adherent cells, 0% FBS for 3D and sphere cultures). For viability assay, cells were treated for 72hrs with DMSO, MRT-92, MZ1, JQ1, or their combinations in 1% FBS at the following concentrations: SSM2c: MRT-92 at 250nM and MZ1 at 125nM; MeWo: MRT-92 at 300nM and MZ1 at 125nM; A375: MRT-92 at 250nM and MZ1 at 250nM. Data are expressed as fold percentage of vehicle (DMSO) ± SD.

For isobologram, cells were treated for 72hrs in 1% FBS with vehicle (DMSO), MRT-92, MZ1 or combinations at IC50 doses. Cells were then fixed with 4% PFA, stained with a 0.1% crystal violet solution in deionized water plus 20% methanol at RT, and the OD of released crystal violet quantified in a Victor X5 plate reader (PerkinElmer).

**Chromatin immunoprecipitation**

For ChIP experiments, SSM2c (3x10^6^) cells were crosslinked with 1% formaldehyde for 15min followed by quenching with 125 mM glycine. Pellets were resuspended in 1ml cold Farnham lysis buffer (5 mM PIPES pH 8, 85 mM KCl, 0.5% NP-40) supplemented with protease inhibitor cocktail, nuclei collected by centrifugation at 4500rpm for 10min and then resuspended in 300μl cold nuclear lysis buffer (1% SDS, 10 mM EDTA, 50 mM Tris-HCl pH 8) supplemented with protease inhibitor cocktail. Samples was sonicated with a SONOPULS Mini20 Sonicator (Bandelin) at 4°C, cell debris were removed by centrifugation at 14000rpm for 15min, and chromatin diluted with ChIP Dilution Buffer (10 mM Tris-HCl pH 8, 2 mM EDTA, 140mM NaCl, 1% Triton X-100, 0.1% SDS) and incubated overnight with 20μl protein G magnetic dynabeads and 3μg of mouse anti-SOX2 (R&D System, #MAB2018) or normal mouse IgG (Santa Cruz Biotechnology, #sc-2025) antibodies. Primer sequences are listed in Supplementary Table 1. Data are presented as % of input and expressed as fold of IgG control ± SEM. ACTIN promoter was used as negative control.

**Quantitative RT-PCR**

Total RNA was extracted with TriPure Isolation Reagent (Roche Diagnostics), treated with DNase I (Roche Diagnostics) and subjected to reverse transcription with High-Capacity RNA-to-cDNA Kit (Applied Biosystems) according to manufacturer’s instructions. Quantitative real-time PCR was carried out at 60°C using FastStart SYBR Green Master (Roche Diagnostics) in a Rotorgene-Q (Qiagen, Hilden, Germany). Primers were designed by using Primer3. Primer sequences are listed in Supplementary Table 1. The Pearson’s correlation coefficient was calculated for SOX2, BRD4 and GLI1 genes, and the p-values adjusted using Bonferroni multiple-testing correction.

**Western blot and co-immunoprecipitation**

For Western blot, cells were lysed for 20min in cold RIPA buffer (50mM Tris-HCl pH 7.5, 1% NP-40, 150 mM NaCl, 5 mM EDTA, 0.25% NaDOC, 0.1% SDS) supplemented with protease and phosphatase inhibitors. After centrifugation, supernatant was collected as whole cell extract (WCE). Equivalent amounts of protein were resolved by SDS-polyacrylamide electrophoresis in 8-12% gels and transferred by electroblotting to a nitrocellulose membrane.

For protein co-immunoprecipitation, cells were lysed in cold IP buffer (0.5%NP-40, 100mM NaCl, 5mM EDTA, 10% glycerol, 50mM Tris-HCl pH7.5) supplemented with protease and phosphatase inhibitors, and supernatant was collected as WCE after centrifugation at 14000rpm for 15min at 4°C. 1mg WCE was diluted with IP buffer to a final volume of 500μl and incubated overnight with 50μl of Protein A/G PLUS-Agarose beads (Santa Cruz Biotechnology, #sc-2003) and 5μg of mouse anti-SOX2 (R&D System, #MAB2018), rabbit anti-BRD4 (Cell Signaling, #E2A7X) or normal mouse IgG (Santa Cruz Biotechnology, #sc-2025). Where indicated, 200μg/ml ethidium bromide (ThermoFisher Scientific) or 25U/ml DNase I (Roche Diagnostics) was added prior to co-immunoprecipitation. Protein-antibody complexes were detected by using SuperSignal West Femto (ThermoFisher Scientific) and imaged with ChemiDocTM Imaging Systems (Bio-Rad).

List of primary antibodies is reported in Supplementary Table 3.

**The Cancer Genome Atlas analysis**

The University of California Santa Cruz Xena platform was used to visualize and analyze transcriptomic and survival data from 477 cases in the TCGA melanoma (SKCM) cohort [1]. Cases were sorted by high and low expression of GLI1 and/or SOX2 according to RNA-seq data associated with the SKCM cohort. Xena was used to generate a Kaplan Meier plot using overall survival as a default. Survival time is measured in days and plots extend to the last time any individual in the plot was known to be alive. Statistical comparison of the two subgroups was carried out using the log-rank test.

**SUPPLEMENTARY REFERENCES**

1. Goldman M, Craft B, Hastie M, Repelcka K, McDade F, Kamath A, *et al*. The UCSC Xena platform for public and private cancer genomics data visualization and interpretation. 2019, bioRxiv 326470 doi: <https://doi.org/10.1101/326470>

**SUPPLEMENTARY FIGURES**

**Supplementary Figure 1. Sequence of the *GLI1* promoter**

UCSC Genome Browser GRCh38/hg38 Assembly

>hg38_dna range=chr12:57457553-57459785 5'pad=0 3'pad=0 strand=+ repeatMasking=none

Position: from -2585 to +3289 bp from TSS (see Fig. 1A)

TGATTCTTCTGCCTCAGCCTCCCGAGCAGCTGGGATTACAGGCGCCCACTAATTTTTGTATTCTTAGTAGAAACGAGGTTTCAACATGTTGGCCAGGATGGTCTCAATCTCTTGACCTCTTGATCCACCCGACTTGGCCTCCCGAAGTGATGAGATTATAGGCGTGAGCCACCGCGCCTGGCTTATACTTTCTTAATAAAAAGGAGAAAGAAAATCAACAAATGTGAGTCATAAAGAAGGGTTAGGGTGATGGTCCAGAGCAACAGTTCTTCAAGTGTACTCTGTAGGCTTCTGGGAGGTCCCTTTTCAGGGGTGTCCACAAAGTCAAAGCTATTTTCATAATAATACTAACATGTTATTTGCCTTTTGAATTCTCATTATCTTAAAATTGTATTGTGGAGTTTTCCAGAGGCCGTGTGACATGTGATTACATCATCTTTCTGACATCATTGTTAATGGAATGTGTGCTTGTATGGTCTTGTGTTACAGTCTTTTTCAGTTTTAATTTCTAATGTGGTGAATTGATAGATGTAAATCATATGAACAAAAGCTCAATGATTTTTTTTTTTTTGAGACGGAGTCTCGCTCTGTCGCCCAGGCTGGAGTGCAGTGGCACAATCTCGGCTCACTGCAACCTCCACCTCCCAGGTTCAAGCGATTCTCCTGCCTCAGCCTCCTGAGTAGCTGGGACTACAGGCCTGCGCCACCATGCCCAGCTATTTTTTGTATTTTTAGTAGAGATGGGGTTTCACCATGTTGGCCAGGCTGGTCTCGGACTCCTGACTTGAGGTGATCCGCCTGCCTTGGCCTCCCAAAGTGCTGGGATTACAGGTCTGAGCCACTGTGCCTAACCTAATGACTTTTAAGAGTATAGAGGAAACCAAAAAGTTTGAGACTCACTGGTCTATAGAACTGGGTGGGGAGAAGAAAGTAAGGTGTTCTAAGAGAGCTCTTCTTGCTGGGCACCGGTGGTCCCAGCTACTTAGGAGGCTAAGGCCGGAAGACCGCTTAAACCCAGGAGTTCGAGGCTATGATCACACTTGTGAATAGCCTCTGCACTCCAGCCTGGGCAAATAGTGAGGCCCCGTCCCAAATAAAAATAAATAAATACATAAATACATAAATAGCTCCTCTGGAAGAAGGGGCTCGAGGCTGGGACAGGAGCATGTGTGGGGTGCCTTTTTTTCAGTGCCCATTAGTCTGGTCTGACTGAGCTGGGTCTCTGACCCTCTGGGGATAACTAGCCTGGGTCAAAGTCCCAGATCTCCCCCTACCTTCACCTTTTCTTTTCCCCCTTGACCCTCAGACTGAACAGTTAACCCACTGACCTTCCACACCCAAGGGGGTGGTTCTTGGAAGCAGAGCTAGGATGTGGGAGGTCTGCCTGTGGGGTTGAAAAAAAGGGGAGAGGGTGCCCTTTTTCCTTGTCATGCTTCCTCCTCTTTCTCATAAAAATCAGAGACTGAAATGCTGCCCCTCCCTTATATCCGGTCACGATGGCAATGCAAATCTAAAGAGGCAGGGCACTTCCCTGTCAGGCAGTACCGCTGGGCATAGCAACCTCTGCCTCTCCGTTTCTCAGAGCTCACATATCCACCTCCTGGGCTTTTAAGTGGGCTTTAGTGAGGGGCTCCTCCTTCAACTGGGCTCCTCCTTCAGTTCCCCAGCTCTTCTGCTTCGACTCCGAGCGGGTGTCATGTGTGAGAACGGCCAGCAGAGGGAGCAGAAAGCCTGGAAGAGCAGCTAGAGCCTGCAGTGACGTGGTGCGGAGGGGCGGCACCCTCCAGAACTTCGAGACGTAGAGCCGGGGTTCTAGGGAAAGGGGCTTCAGTCCCAGGGCTCCTTGGTGACCTCGTGAACCACACCCTGCACCCAGAGCCTCAGCCGCTGCTCCTTGCTTTTATGCTCCATAGACTCCTCACCTTCTTCCAGAGCCCCCAACCCAACTTGATTTGCCCCAAACCGCAACTCTGTCCCGGCCGCTGCAAGTTCCATCCAAAGGGTGAGGCCTGCAGATAAACCACAGGATGGCAGAATGCTCAGTTAGCACCAACCAAAGGCGACTACCCTACCTCCACTATTATCGTTCTCGGTTGAACTTCTCCCCCTGCCCCGCAATATTTTCCTCAATCTGGTTGTCGGGGCCTCTTTGGGGCCAGCCGATCCAGAAATCCAAGCCGGGATTTAGTACTCACCAACAGCAGCGTGTTCAGCCGGGGCGGGGGGGGGGGCG

**Supplementary Figure 2.** qPCR (**a**) and Western blot (**b**) of SSM2c, MeWo and A375 melanoma cells transduced with LV-c or LV-shSOX2.2. ACTIN was used as loading control (**b**) (n=3). *, p<0.05; **, p<0.01.

**
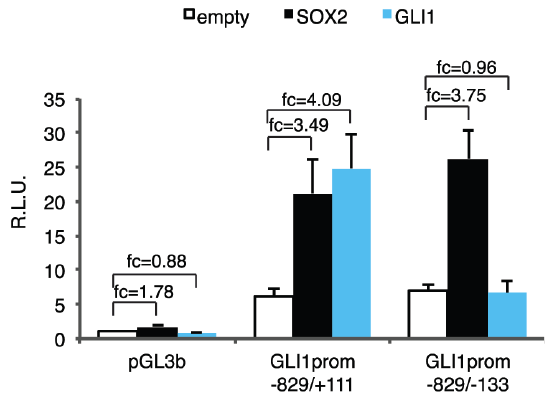
**

**Supplementary Figure 3.** Quantification of dual-luciferase assay in SSM2c cells showing the levels of activity of the -829/+111bp and the -829/-133bp fragments of *GLI1* promoter compared to that of empty vector (pGL3-b) after SOX2 (black bars) or GLI1 (blue bars) overexpression (n=3). Data are represented as mean ± SEM. Fold change (fc) are indicated.

**
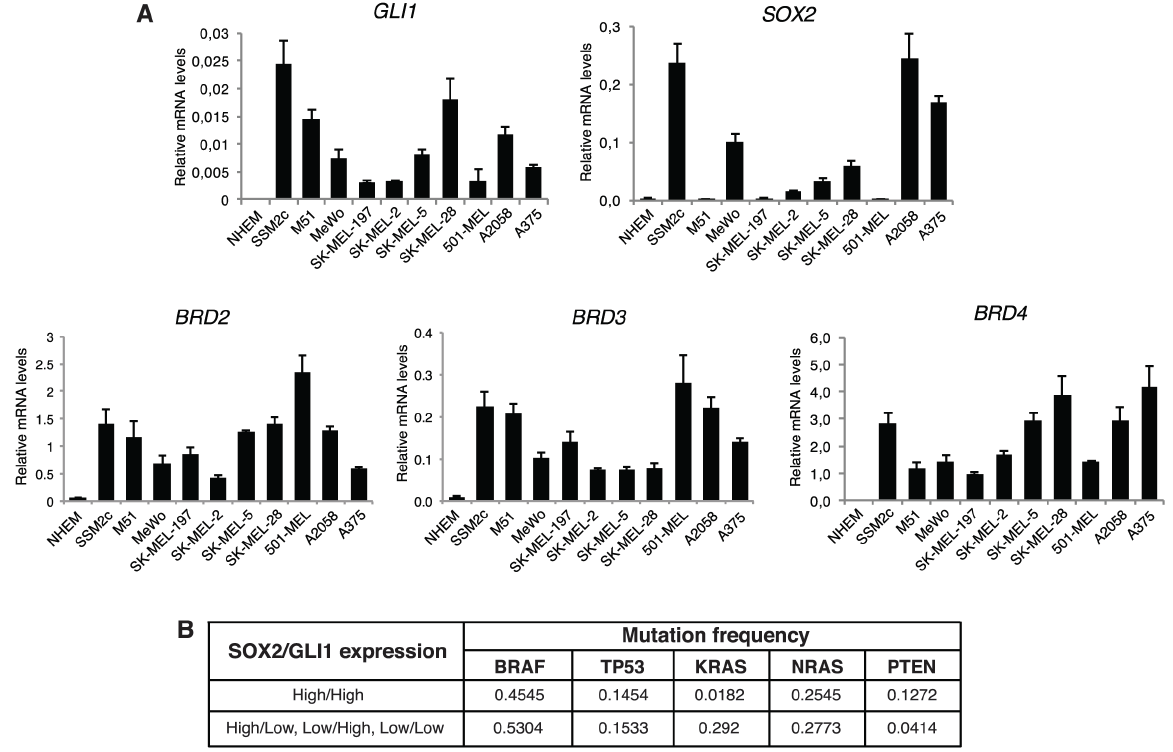
**

**Supplementary Figure 4. Expression of *SOX2, GLI1* and *BRD* members in melanoma cells and mutation frequency in human melanoma samples. a** qPCR of *GLI1*, *SOX2, BRD2, BRD3* and *BRD4* in normal human epidermal melanocytes (NHEM) and in a panel of human melanoma cell lines. Gene expression was normalized relative to *TBP* and *HPRT* housekeeping genes and expressed as mean ± SEM. **b** Mutation frequencies of driver mutations in melanoma cases. TCGA melanoma cohort was separated into subgroups based on expression levels of SOX2 and GLI1. Mutation frequency was calculated for each subgroup as number of cases with mutation/total cases in subgroup.

**Supplementary Figure 5. Specificity of MZ1 towards BRD4. a** Representative Western blot of BRD2, BRD3 and BRD4 in SSM2c, MeWo and A375 melanoma cells treated with increasing concentrations of MZ1 for 72hrs. HSP90 was used as loading control. Quantification of BRD2, BRD3 and BRD4 proteins, expressed as relative ratio of each BRD/HSP90, is shown in blue. **b** Western blot analysis of BRD2, BRD3 and BRD4 in SSM2c melanoma cells transduced with LV-c or LV-shBRD2, and LV-c or LV-shBRD3. HSP90 was used as loading control. **c** Histogram of SSM2c melanoma cell viability in cells transduced with LV-c, LV-shBRD2 or LV-shBRD3 and treated with DMSO or increasing concentrations of MZ1 (n=3). **d** Western blot analysis of BRD2, BRD3 and BRD4 in A375 melanoma cells transduced with LV-c or LV-shBRD2, and LV-c or LV-shBRD3. ACTIN was used as loading control. **e** Histogram of A375 melanoma cell viability in cells transduced with LV-c, LV-shBRD2 or LV-shBRD3 and treated with DMSO or increasing concentrations of MZ1 (n=3). Data are presented as mean ± SD (**c**, **e**). *, p<0.05; **, p<0.01; ***, p<0.0001.

**
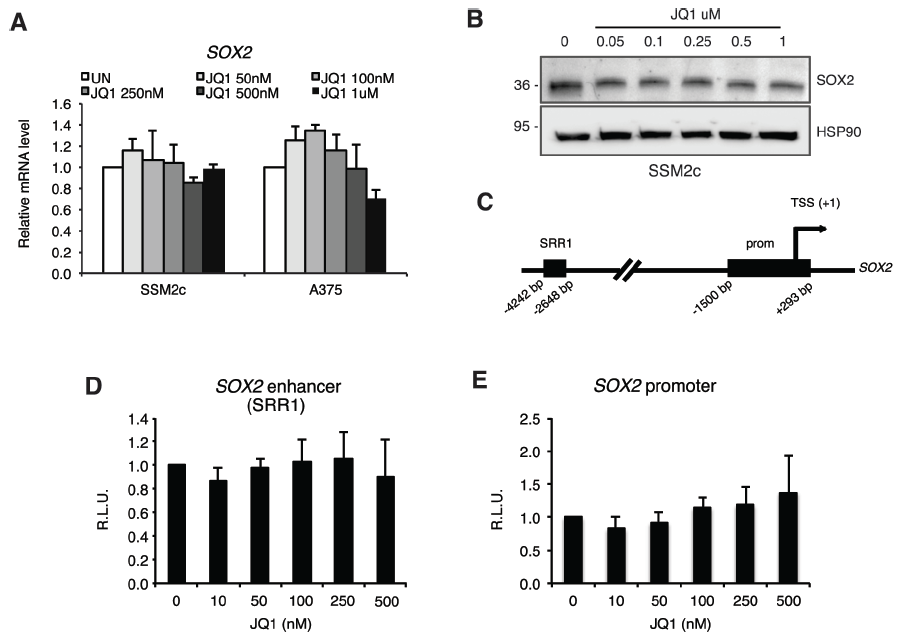
**

**Supplementary Figure 6. BRD4 does not affect SOX2 expression in melanoma cells. a** qPCR of SOX2 in SSM2c and A375 treated with increasing doses of JQ1. Gene expression was normalized relative to *TBP* and *HPRT* housekeeping genes and expressed as mean ± SEM. **b** Representative Western blot of SOX2 in SSM2c treated with increasing doses of JQ1. HSP90 was used as loading control. **c** Schematic representation of SOX2 regulatory regions (SRR1, distal enhancer; prom, promoter). **d,e** Quantification of dual-luciferase assay in SSM2c transfected with SOX2 enhancer (**d**) or promoter (**e**) and treated with increasing doses of JQ1 (n=3). Data are presented as mean ± SEM.

**Supplementary Figure 7. Effects of SOX2 and BRD4 silencing on *GLI1* expression.**

**a, b** qPCR analysis of BRD4, SOX2 and GLI1 in SSM2c (**a**) and A375 (**b**) cells transduced with LV-c, LV-shBRD4, LV-shSOX2, or their combination. Data are presented as mean ± SEM (n=3).

**
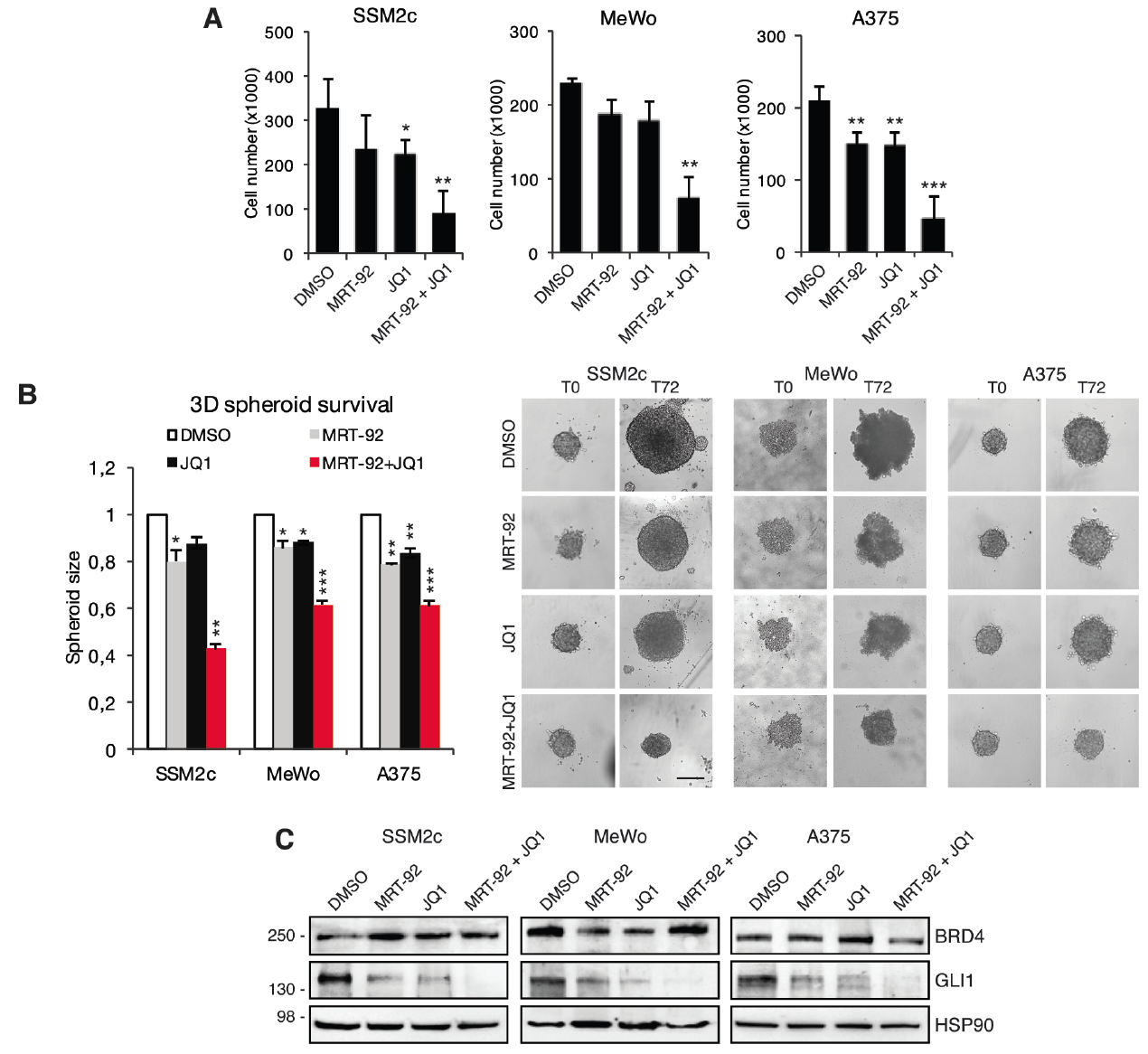
**

**Supplementary Figure 8. Combined treatment with MRT-92 and JQ1 inhibits melanoma cell growth in 2D and 3D. a** SSM2c, MeWo and A375 cell viability after 72hrs of treatment with MRT-92, JQ1 or combination at the following concentrations: SSM2c: MRT-92 at 125nM and JQ1 at 100nM; MeWo: MRT-92 at 300nM and JQ1 at 100nM; A375: MRT-92 at 125nM and JQ1 at 250nM (n=3). **b** Quantification of 3D spheroid diameter and representative images of spheroids at the optimized seeding densities (T0) or 72hrs after treatment with vehicle (DMSO), MRT-92, JQ1 or combination at the concentrations indicated in (**a**) (n=3). Scale bar=200 µm. **c** Western blot of BRD4 and GLI1 in melanoma cells after treatment with MRT-92, JQ1 or combination for 72hrs. HSP90 was used as loading control. Data are presented as mean ± SEM. *P* values were calculated by one-way ANOVA with Tukey’s test. *, p<0.05; **, p<0.01; ***, p<0.0001.

**SUPPLEMENTARY TABLES**

**Supplementary Table 1. List of primers used for qPCR.**

| **Primer** | **Sequence (5’ to 3’)** |
| --- | --- |
| GLI1promA-F | GGTCCTGGGGGTGCAATAAG |
| GLI1promA-R | GCCCCTCACCTCCCTTCTAT |
| GLI1promB-F | TCTCCCCCTACCTTCACCTT |
| GLI1promB-R | AGGAGGAAGCATGACAAGGA |
| GLI1promC-F | GGCCGTGTGACATGTGATTA |
| GLI1promC-R | AGCGAGACTCCGTCTCAAAA |
| ACTINprom-F | TCGAGCCATAAAAGGCAACT |
| ACTINprom-R | CTTCCTCAATCTCGCTCTCG |
| BRD2-F | CCCTTCTGGCTTTGGACCTT |
| BRD2-R | TGCCGCTTCTCATCGTAACT |
| BRD3-F | GTGGTGAAGACGCTCTGGAA |
| BRD3-R | AAGCTTGGGCCATTAGCACT |
| BRD4-F | TGACAGCGAAGACTCCGAAA |
| BRD4-R | GTGATGATGGTGCTTCTTCTGC |
| TBP-F | CAACAGCCTGCCACCTTAC |
| TBP-R | CTGAATAGGCTGTGGGGTC |
| GLI1-F | CCCAGTACATGCTGGTGGTT |
| GLI1-R | GCTTTACTGCAGCCCTCGT |
| HPRT-F | GCCAGACTTTGTTGGATTTG |
| HPRT-R | CTCTCATCTTAGGCTTTGTATTTTG |
| SOX2-F | GAGCTTTGCAGGAAGTTTGC |
| SOX2-R | GCAAGAAGCCTCTCCTTGAA |

**Supplementary Table 2. List of primers for cloning or mutagenesis.**

| **Primer** | **Sequence (5’ to 3’)** |
| --- | --- |
| GLI1prom-829/+111 F | CGGCACCCTCCAGAACTTCG |
| GLI1prom-829/+111 R | CCCCTCAGGCCCCCTCCC |
| GLI1prom-829/-133 F | CGGCACCCTCCAGAACTTCG |
| GLI1prom-829/-133 R | CGCGAGAAGCGCAAAC |
| GLI1prom-584/-133 F | CTGCAGATAAACCACAGGATG |
| GLI1prom-584/-133 R | CGCGAGAAGCGCAAAC |
| Mut1-F | TGCTCAGTTAGCACCAAGGAAAGGCGACTACCCTAC |
| Mut1-R | GTAGGGTAGTCGCCTTTCCTTGGTGCTAACTGAGCA |
| Mut2-F | GCCAGCCGATCCAGAAATCGGAGCCGGGATTTAG |
| Mut2-R | CTAAATCCCGGCTCCGATTTCTGGATCGGCTGGC |
| Mut3-F | AGCGGCTGGAGAGAGAAAAAGTTTTCCCAAAAGGGAAAAAAAAAGTTTG |
| Mut3-R | CAAACTTTTTTTTTCCCTTTTGGGAAAACTTTTTCTCTCTCCAGCCGCT |

**Supplementary Table 3. List of primary antibodies used for Western blotting.**

| **Antibody** | **Source** | **Cat. No.** | **Company** |
| --- | --- | --- | --- |
| SOX2 | Mouse | MAB2018 | R&D System |
| GLI1 | Mouse | 2643 | Cell Signaling |
| γ-H2AX | Mouse | 05-636 | Merck Millipore |
| BRD2 | Mouse | sc-393720 | Santa Cruz Biotechnology |
| BRD3 | Mouse | sc-81202 | Santa Cruz Biotechnology |
| BRD4 | Rabbit | E2A7X | Cell Signaling |
| PARP-1 | Rabbit | 9542 | Cell Signaling |
| β-ACTIN | Mouse | sc-47778 | Santa Cruz Biotechnology |
| HSP90α/β | Mouse | sc-13119 | Santa Cruz Biotechnology |
| IgG | Mouse | sc-2025 | Santa Cruz Biotechnology |
